# A framework for the estimation of the proportion of true discoveries in single nucleotide variant detection studies for human data

**DOI:** 10.1371/journal.pone.0196058

**Published:** 2018-04-25

**Authors:** Nik Tuzov

**Affiliations:** Partek Incorporated, Saint Louis, Missouri, United States of America; CNR, ITALY

## Abstract

Any single nucleotide variant detection study could benefit from a fast and cheap method of measuring the quality of variant call list. It is advantageous to be able to see how the call list quality is affected by different variant filtering thresholds and other adjustments to the study parameters. Here we look into a possibility of estimating the proportion of true positives in a single nucleotide variant call list for human data. Using whole-exome and whole-genome gold standard data sets for training, we focus on building a generic model that only relies on information available from any variant caller. We assess and compare the performance of different candidate models based on their practical accuracy. We find that the generic model delivers decent accuracy most of the time. Further, we conclude that its performance could be improved substantially by leveraging the variant quality metrics that are specific to each variant calling tool.

## Introduction

Identifying single nucleotide variants (SNV) is a major application of next-generation sequencing. SNV calling is a multistep process that is not over once a variant caller is invoked. In particular, every variant caller allows the user to specify at least one parameter to adjust the sensitivity of the call list by imposing a threshold on the variant quality score denoted by QUAL in Variant Call Format, VCF. In addition, a variant caller usually produces a number of variant-level statistics (depth, strand bias, average base and mapping quality, to name a few) that are meant to be used for downstream variant filtering to adjust the call list quality further.

While it is possible to come up with some reasonable filtering thresholds, the ways of observing how different filtering settings impact the quality of the call list (if at all) are fairly limited. Such approaches as verifying the result by applying Sanger sequencing or SNV array are expensive. Obtaining variant calls starting from physical samples can be expensive, too. Hence, the researcher might decide to reuse the variants from previous studies. In that case, one has access only to the variant call lists from a database (in VCF format), facing the necessity of estimating and adjusting the call list quality based just on the content of available files [1].

In particular, a researcher would be very interested in estimating a proportion of true variants in the call list (positive predictive value, PPV, aka Precision). That quantity can be measured explicitly but only if the dataset is a gold standard where the true and false variants are known in advance. The gold standard approach has been successfully used for comparing variant calling pipelines or parts thereof (most importantly, different variant callers) in [2, 3, 4, 5, 6].

However, in a real project the dataset of interest is not a gold standard. Even if experimenting with a gold standard results in a recommended variant calling pipeline, one cannot be sure how, in quantitative terms, that pipeline will work for a different dataset. For instance, if a given pipeline resulted in 85% PPV on a gold standard dataset, how likely is that to be reproduced on a new dataset? Apparently, some deviation will occur. If so, is it possible to specify a prediction interval for a future PPV?

A simple linear model to estimate PPV as a function of transition/transversion ratio, Ti/Tv, was proposed in [7], but, to the best of our knowledge, it has not been developed much. In practice, the most popular usage of Ti/Tv is a rule that for a whole genome sequencing (WGS) or whole exome sequencing (WES) call list to be of high quality, the Ti/Tv should be around 2.0 or 3.0 respectively [8]. Apart from being crude, this rule aims for the call list with close to zero proportion of false positives (FP), even if that implies a very low sensitivity. In other words, the rule of thumb is not going to prevent one from failing to identify a sizable proportion of true positives (TP) as such. It works fine if, indeed, the researcher’s goal is to maintain a very low FP proportion at any cost, but that does not have to be the case. For instance, if one tries to experiment with producing variants by intersecting call lists from two or more variant callers, one can choose to tolerate a higher FP proportion for each variant caller because otherwise the final intersection-based call list would be too small.

One more application of PPV estimation is the variant quality score recalibration [7]. Recalibration aims to improve the quality score by taking variant annotations into account after an initial variant call list has been obtained. An important input for the recalibration algorithm in [7] is the set of “bad” (FP) variants. A possible way to specify it is to suggest that (1 –PPV) % of variants with the lowest QUAL score are “bad”.

One possible reason why few researchers (one exception is [1]) looked into extending the model in [7] is the belief that the variant calling results depend too much on the sequencing platform, exome capture kit, aligner, and variant caller [9, 3, 1, 6]. That appears to be true, but, as far as we know, it only means that, given different pipelines, the generated variant call sets might not intersect too much. However, using Ti/Tv as an example, what if the relationship between PPV and Ti/Tv is about the same for the most popular variant callers? In that case, it should be possible to build a model to predict PPV based on Ti/Tv without having to adjust it for a specific variant caller and other factors mentioned above. To the best of our knowledge, that option has never been investigated using a strict quantitative approach.

In this paper, we are looking into a possibility of building a model that can estimate PPV for a human SNV study (indels are not considered). The estimation method is based on a few variant quality statistics available for any variant caller. Our approach is to use several gold standard data sets to learn the relationship between PPV and the quality statistics. Whether our approach is viable is immediately clear because we are able to assess the accuracy of the candidate models in explicit, practically meaningful terms. As a result, we are able to come up with a fairly accurate model that could be improved further in the future.

## Materials and methods

### Data

Our analysis is based on the “Genome in a Bottle” gold standard sample NA12878 [10]. The particular datasets come from two sources. First, [6] provide access to two Illumina datasets: WES 50X (Nextera exome capture kit with 62Mb target region) and WGS 30X. Second, we used Genome Comparison & Analytic Testing tool that was publicly available online and allowed the user to download two WES Illumina datasets, 30X and 150X. To the best of our knowledge, they were generated using TrueSeq exome capture kit with 45 Mb target region. We made the TrueSeq data publicly available [11] because the corresponding website is no longer functioning.

The datasets from [6] come in BAM format (aligned with BWA), whereas TrueSeq datasets are downloaded in FASTQ format and aligned with BWA in Partek Flow. After that, duplicates are removed using Filter Alignments task and the variant calls are obtained using Samtools and Freebayes in Partek Flow. For WES data, the calls are restricted to the corresponding Nextera or TrueSeq target regions.

All of the calls are restricted to chromosomes 1–22 and X (NA12878 is derived from a female). That is done because we intend to use Ti/Tv as a predictor in the model, and mitochondrial regions, Y chromosome, and X chromosome in males are associated with abnormally high Ti/Tv ratios [12]. For such regions, a separate model is needed.

Given the methodology outlined in [5, 13, 14], we developed Variant Validation task in Partek Flow. The Variant Validation task functionality and output are similar to that of formerly available Genome Comparison & Analytic Testing tool. Using any gold standard dataset as input, the task produces a set of comprehensive performance evaluation metrics for a variant calling pipeline: sensitivity, specificity, PPV, and many others.

By using different datasets, variant callers, and tweaking the variant filtering options such as quality (QUAL) and depth (DP) thresholds, we obtain a large number of variant call lists. Each list is fed to Variant Validation task that measures the observed values of TP, FP, PPV, and different statistics that might be predictive of PPV. As a result, we obtain a training data set where each observation is derived from a distinct call list and we use the data set to discover a relationship between PPV and the predictors.

### Composition of predictor pool

Along with Ti/Tv, the heterozygous/homozygous ratio, Het/Hom, is used for quality control [15, 16] where higher values of Het/Hom are associated with lower quality call sets. According to [17], in theory Het/Hom ratio should be 2.0 for WGS, and no possible dependence on the ancestry (race) is mentioned. For WES, no expected value is specified, except for stating that it should be “significantly lower”. Also, [16] report that Het/Hom thresholds for WES data are determined by “historical values” without disclosing what actual thresholds are used. According to [12], Het/Hom ratio is very much influenced by ancestry (which is also confirmed by [18]), but not influenced by the genomic region (exonic vs non-exonic). It therefore appears that the usage of Het/Hom as a quality control metric is not as well understood as the usage of Ti/Tv. Correspondingly, part of our agenda is to quantify the contribution of Het/Hom ratio by including it in the model.

In addition, [12] suggest that Het/Hom is possibly “orthogonal” to Ti/Tv: Ti/Tv is related to type of genomic region, GC content, functionality (% of synonymous SNVs), but not ancestry. Het/Hom is related to ancestry, but not to the type of region, GC content, or functionality. The possible “orthogonality” of Het/Hom and Ti/Tv is another reason for including the former in the model. Also, each variant call set is characterized by median depth (MedDp), proportion of variants with depth below five (DpLt5), and a binary indicator (WES_Indicator) that is equal to 1 for WES study and 0 for WGS. As a result, we have four quantitative (Ti/Tv, Het/Hom, MedDp, DpLt5) and one categorical (WES_Indicator) predictor.

We perform the initial selection of second order terms as follows. First, we include the squared values of all four quantitative predictors to account for a possibility of a curvilinear relationship between them and PPV. Second, given the information outlined above, it is apparent that the relationship between PPV and such predictors as Ti/Tv and Het/Hom is probably different in WES and WGS data sets. For that reason, we include interaction terms TiTv * WES_Indicator and Het/Hom * WES_Indicator. While there is no prior evidence that a similar reasoning applies to depth statistics, we include the corresponding interactions, MedDp * WES_Indicator and DpLt5*WES_Indicator, as well. Sensitivity as a function of depth is different for homozygous and heterozygous SNVs [19], which prompted us to add the MedDP * Het/Hom ratio term. Finally, the interaction of Ti/Tv and depth metrics were added for exploration purposes. Centering to the mean was performed prior to computing all of the second order terms. In total, the first and second terms span 18 scalar parameters, including the intercept.

A fairly obvious question is why the variant quality score itself (QUAL) is not used as one of covariates. VCF standard implies that regardless of the variant calling method used, the meaning of QUAL value is exactly the same (Phred-scaled probability that the call in ALT column is wrong). However, we are still not perfectly sure that QUAL values generated by Samtools and Freebayes are directly comparable, hence QUAL is excluded.

### Model building procedure

An SNV call list of size N can be seen as a result of N “trials” with binary outcomes where “success” corresponds to a true variant (TP) and “failure” corresponds to a false variant (FP). The observed TP proportion, or PPV, is defined as

$$PPV\;=\;\frac{TP}{N}\;=\;\frac{TP}{TP+FP}$$ (1)

The counts of TP and FP can be obtained explicitly for a gold standard data set, and we can train a model where the probability of “success” is a function of predictors. It is therefore understandable why we first tried fitting a Binomial regression model. However, we found that Binomial model suffers from a severe overdispersion problem (results not shown), and for that reason we switched to Beta-binomial regression. Under Beta-binomial distribution, the count of successes, Y, in N trials has the following mean and variance:

E[Y]=N∙μ (2)

$$Var[Y]\;=\;N\mu (1-\mu )\times \frac{\left(1+N\sigma \right)}{1+\sigma }$$

Here μ is the probability of success and σ is the dispersion parameter. When the latter is equal to 0 (no overdispersion), the distribution of Y is reduced to Binomial. Parameterization (2) is used in gamlss() procedure in R that we employ for model fitting. The link between (2) and a more conventional (α,β) parameterization is:

$$E[Y]\;=\;\frac{N\alpha }{\alpha +\beta }$$ (3)

$$Var[Y]\;=\;\frac{N\alpha \beta (\alpha +\beta +N)}{{\left(\alpha +\beta \right)}^{2}(\alpha +\beta +1)}$$

$$\alpha \;=\;\frac{\mu }{\sigma };\;\beta \;=\;\frac{1-\mu }{\sigma }$$

To incorporate the covariates into (2), the following inverse link functions are used:

$$\mu \;=\;\frac{exp(x^{\prime }\beta )}{1+exp(x^{\prime }\beta )}$$ (4)

$$\sigma \;=\;exp(z^{\prime }\gamma )$$

where x and z are the vectors of covariate values and β and γ are the estimated regression coefficients. The covariates that describe μ and σ may or may not be the same, but in this study z is always a subset of x. Denoting the lengths of β and γ by p and q, we will refer to the model as (p, q) below.

We generate the training data (the values of Y, N, and x) as follows. For instance, we use Nextera data set, apply Samtools with sensitivity 0.99999 and filter the output with DP threshold of 5. That results in 60752 SNV calls, of which 37627 have genotype that is different from the reference. Partek Flow Variant Validation task reports that 37627 calls consist of 35600 TP and 2027 FP and it also reports the values of Ti/Tv, Het/Hom, MedDp, and other summary quality statistics for 37627 calls. In terms of model (1–4), N is equal to 37627, Y is equal to 35600, and the values of Ti/Tv, Het/Hom, etc, are put in the vector x. The values of Y, N, and x constitute a single observation for model (1–4). By varying the data set, variant caller, and filtering thresholds, we obtain about 500 of such observations.

The parameter μ is the expected PPV: if we were to take a large number of variant call sets that have the same covariate pattern x, and then take an average of PPV across the call sets, we should expect to get a value close to μ. However, here we are interested in PPV for a particular call set, an “observed” rather than “expected” PPV. In other words, we would like to obtain a prediction interval for the future observed value of random variable Y/N. In order to do that, we assume that Y/N follows a Normal distribution whose mean and variance can be easily obtained from (2) and then used for constructing a 95% prediction interval. We do not take into account the uncertainty of estimating the regression coefficients β and γ because we assume it to be relatively low due to a sufficient sample size. Such approach will perform well only if the point estimates of β and γ are very close to their true (population) values and the Normal approximation for the distribution of Y/N is adequate.

A prediction interval is constructed for each point in the training data set by using a leave-one-out approach. For a given point, the predicted values of μ and σ are obtained from a model fitted on the data from which the point of interest is excluded. We then compute the actual coverage as the proportion of observations found inside the respective prediction intervals. The actual coverage is close to the nominal 95% for all the models considered below, suggesting that the Normal approximation works well.

Our approach is to start with a “global” model containing the largest possible number of terms. After that we apply backward elimination based on the p-value but the stopping rule is based on Akaike Information Criterion, AIC, [20], rather than on a rigid p-value cutoff such as 0.05. Therefore, backward elimination is essentially used as a tool for defining a fairly small model pool in an adaptive manner.

**Fig 1:**
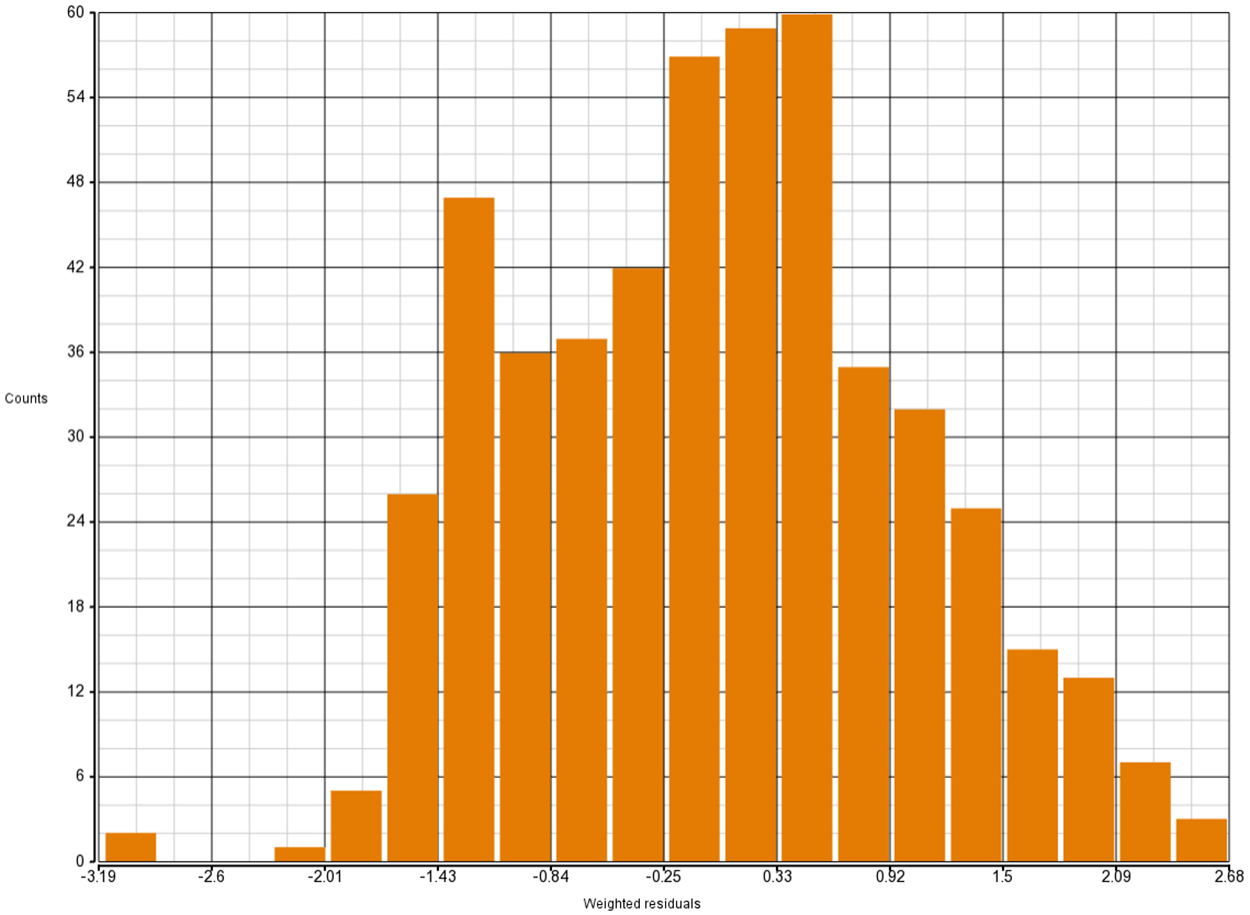
Weighted residuals for model (11, 6) from Table 1.

**Fig 2:**
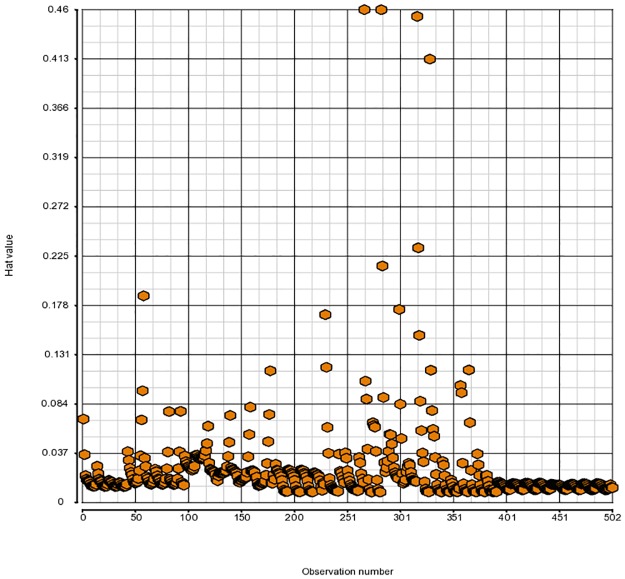
Hat values for model (11, 6) from Table 1.

**Fig 3:**
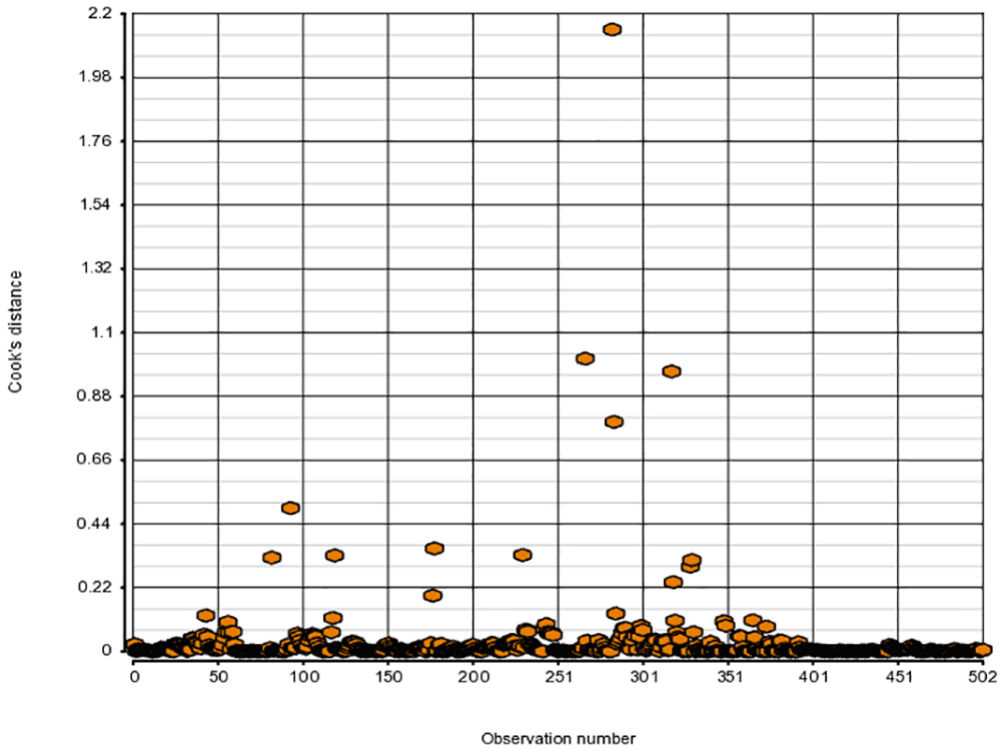
Cook’s distances for model (11, 6) from Table 1.

It would be unwise to apply our model selection procedure if in fact our final model and inference are defined by a few unduly influential observations. To avoid that, we first look at weighted residuals from Beta-binomial regression. Even though the weighted residuals do not follow Normal distribution, they possess the approximate property of having mean 0 and standard deviation 1 which allows us to catch major irregularities, if any (Fig 1). In addition, the hat values identify outliers in the covariate space and Cook’s distances point to observations that exert a large influence on the fitted surface. How to compute those quantities for Binomial regression is explained in detail in [21]. We use that approach to obtain similar metrics for Beta-binomial regression, although we have to rely on Binomial hat values since Beta-binomial hat values are not available in R. The hat values and Cook’s distances we use can provide only partial information about the identity of problematic points in Beta-binomial model (Figs 2 and 3).

**Fig 4:**
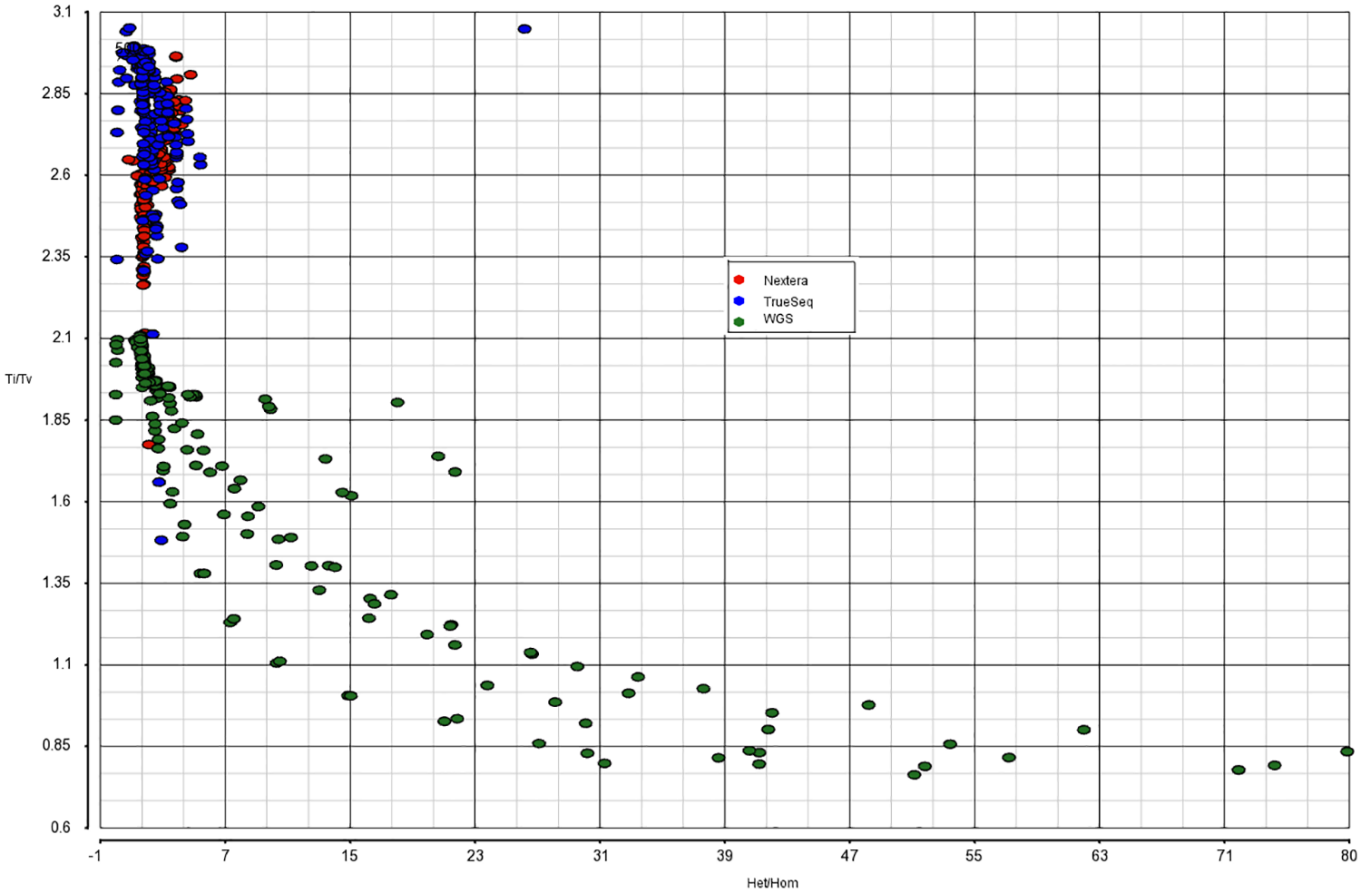
Relationship between Ti/Tv and Het/Hom. Red, blue and green dots denote Nextera, TrueSeq, and WGS observations, correspondingly. Here one can see an outlying blue point obtained with TrueSeq and Freebayes.

Once suspicious points are detected, we fit the regression surface without them to see what kind of impact they have on the p-values and fitted values. As a result, we are able to identify some points that are located too far from the majority of points in the covariate space, but that are acceptable otherwise. Our remedial measure is to tweak the variant filtering thresholds and run Variant Validation task a few more times to collect more points to fill in the gaps in the covariate space. The only truly problematic outlier we have to delete is a point generated by Freebayes for WES TrueSeq data. At a high level of QUAL cutoff (about 215), the proportion of homozygous variants in the call set drops precipitously for some reason, and we end up with an abnormally high Het/Hom ratio (Fig 4).

For model (11, 6) the largest prediction interval, PI, for PPV is 44.45% wide. Therefore, we try to improve the solution by experimenting with the following family of inverse link functions for the variance part of the model:

σ=(z′γ)1λ (5)

**Fig 5:**
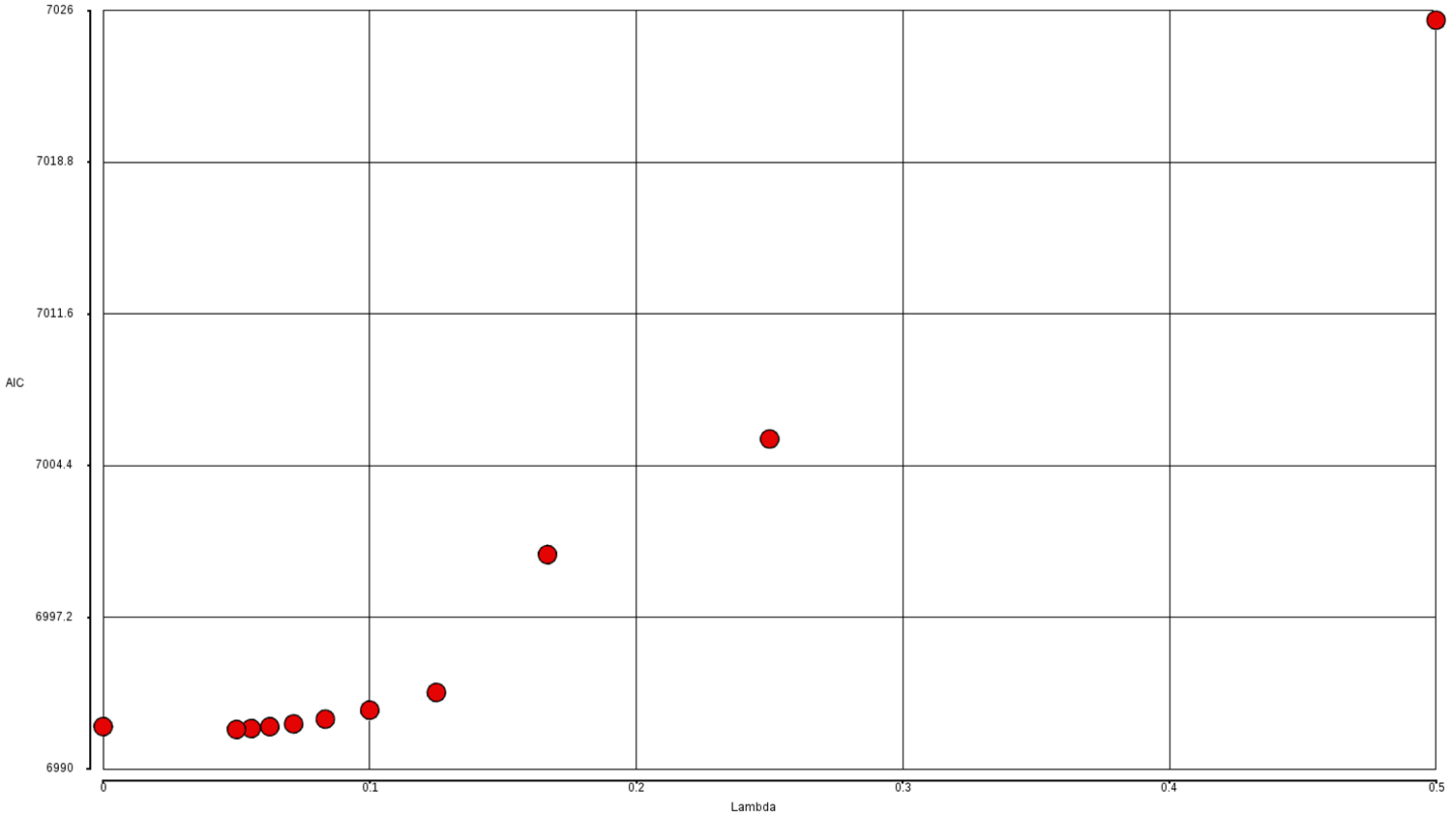
Relationship between AIC and lambda for model (11,6). AIC values for model (11, 6) are plotted against the parameter lambda used in the variance link function in formula (5). The value of lambda equal to zero corresponds to the log link function in formula (4).

Formula (5) employs a Box-Cox type of transformation where the ratio 1/λ goes through a sequence of positive integer values starting from 2. As λ approaches zero, (5) becomes equivalent to the log link function for σ in formula (4). We use AIC to determine the best λ and the results suggest that the log link function is appropriate (Fig 5). The data and R code used for generating the figures and tables in this paper are available in S1 and S2 Datasets and S1 File.

### Model performance evaluation

In order to measure the practical value that can be added by a model, as well as a practical difference between a few competing models, we look at the length of PI for the estimated PPV. It is true that the more parameters we include in the model, the better the fit and the shorter the prediction intervals are. Even though our model selection procedure is data driven, we assume that thanks to a large sample size and the usage of AIC a gross overfitting is avoided and the prediction intervals are more or less representative of what can take place out of sample.

**Fig 6:**
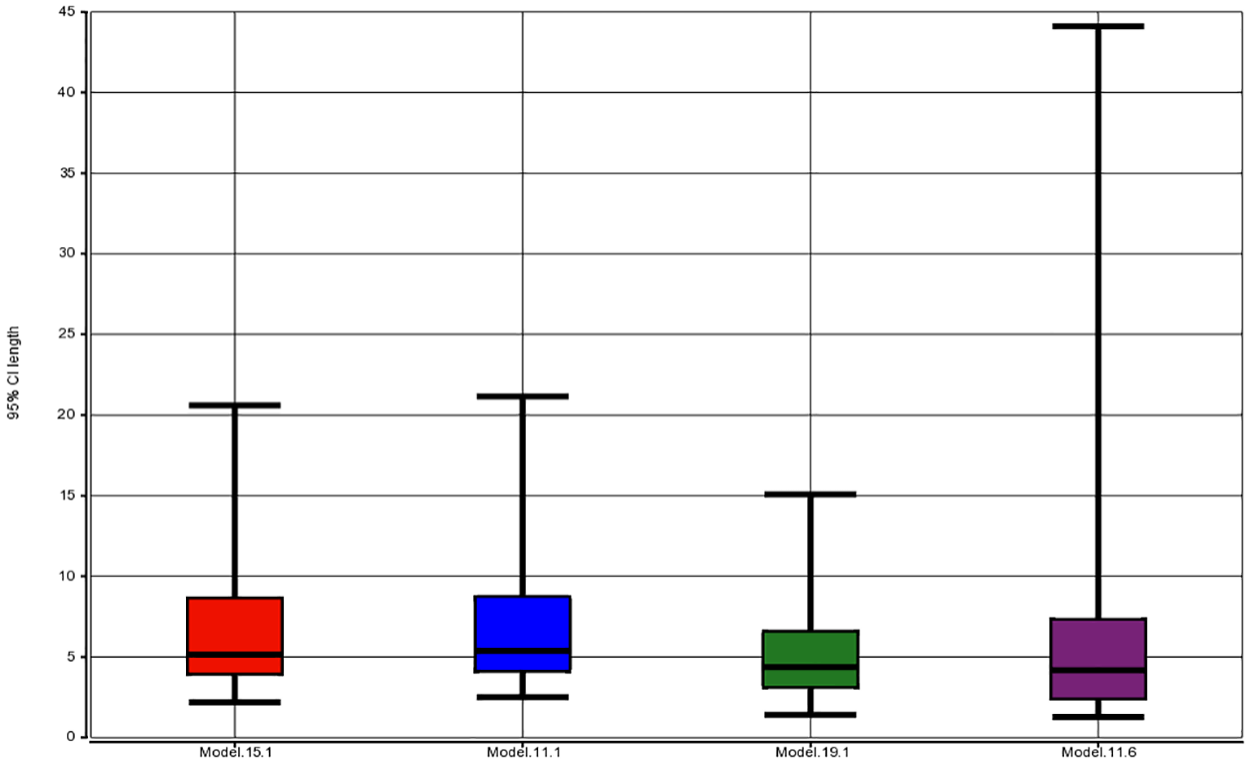
Distribution of the length of 95% PI for PPV. Because PPV is a proportion, the PI length is measured in %. A box plot of length distribution is provided for each model from Table 1.

For each point in the training dataset, we obtain a 95% PI for PPV, compute its length (in percent) and then construct a five-number summary of the lengths. The total number of computed lengths is equal to the number of points in the training dataset, which is about 500. We expect the models that have much higher practical value to result in much tighter PIs which should be clearly visible in the five-number summary (Table 1) and the respective box plot (Fig 6).

**Table 1:** Comparative performance of four candidate models.

(p, q)	AIC	95% PI for PPV, length summary, %					PI coverage, %
		Min	Q1	Med	Q3	Max	
(15, 1)	7219.47	2.18	4.03	5.11	8.61	20.63	94.62
(11, 1)	7230.74	2.49	4.18	5.37	8.71	21.18	95.02
(19, 1)	6932.41	1.40	3.19	4.37	6.48	15.07	93.03
(11, 6)	6992.03	1.22	2.49	4.20	7.19	44.45	95.42

The values p and q denote the number of parameters in the mean and variance part of the model (formula (4)). The five number summary is for the length of 95% prediction interval, PI, for PPV. In particular, for model (11, 6) the length ranges from 1.22% to 44.45%, with the median length of 4.20%. The last column reports the actual coverage of 95% PI.

From the formula (2) we see (assuming for the sake of simplicity that σ is constant) that the variance of response is the highest at *μ* = 0.5 and goes to zero as μ approaches 1. All other things being equal, the length of PI will decrease as PPV approaches 100%, which is advantageous. For instance, for model (11, 6) in Table 1 the median and maximal lengths of PI are 4.20% and 44.45%. If we restrict the summary only to the cases where the point estimate of PPV is over 95%, the median and maximum lengths become 3.16% and 15.40%.

## Results

### Het/Hom ratio has low marginal contribution

**Fig 7:**
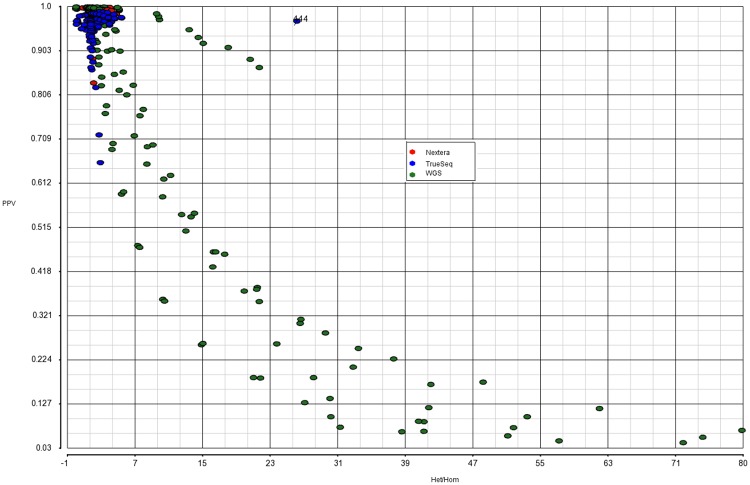
Relationship between PPV and Het/Hom.

**Fig 8:**
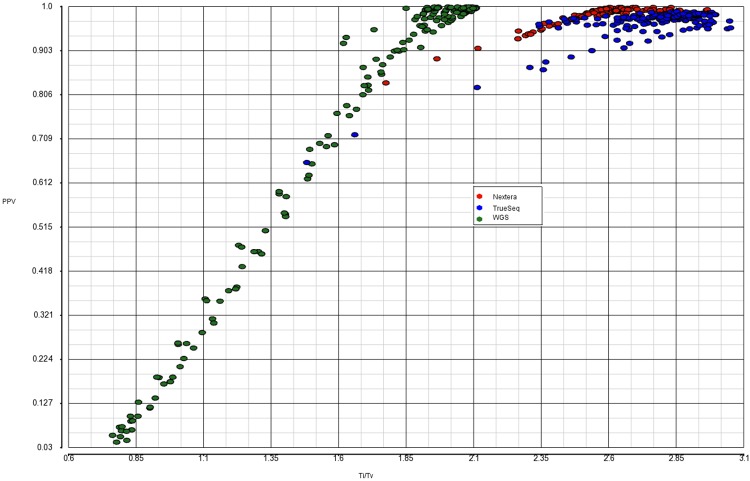
Relationship between PPV and Ti/Tv.

Figs 4, 7 and 8 provide a visual representation of the training dataset, including outliers(s) that are deleted in the end. As expected, there is a strong positive relationship between PPV and Ti/Tv (Fig 8). Contrary to our expectation of “orthogonality” of Ti/Tv and Het/Hom, there seems to be a fairly strong negative association between them (Fig 4).

Using the method described in Model building procedure section, we start with a global (18, 1) model and after three steps arrive at (15, 1) model reported in Table 1. In order to see whether ignoring Het/Hom makes a sizable practical difference, we repeat the procedure without using the Het/Hom related covariates which gives us the next model, (11, 1). In terms of statistical significance, we obtain strong evidence in favor of retaining Het/Hom: if we compare the two models based on their AIC values (7219.47 with Het/Hom and 7230.74 without), the model that contains Het/Hom has Akaike weight of over 99%.

However, comparing the lengths of prediction intervals for these two models, we see that retaining Het/Hom does not improve the performance by a practically significant amount. In a sense, that is good news: [12] suggest that Het/Hom is associated with ancestry (race) and therefore it is possible that the relationship between PPV and Het/Hom is not the same across different levels of race. In that case, if we want to retain the main effect of Het/Hom in the model, the interaction of Het/Hom and race must also be included. The latter is impossible to do because our training data set is derived from a single individual and there is only one level of race. We continue our investigation without taking Het/Hom into account.

### The preparation kit and variant caller have a sizeable effect

In order to take a peek at whether PPV estimation is influenced by the preparation kit and variant caller we include the corresponding terms in the model. In the previous step, the study type factor has two levels (WGS or WES), but now it has three (WGS, WES-Nextera, WES-TrueSeq), and a new factor with two levels (Freebayes, Samtools) is added. The corresponding model is denoted by (19, 1) in Table 1. Comparing models (11, 1) and (19, 1) we see that the inclusion of new terms results in a visible improvement that is far greater than the contribution of Het/Hom terms. However, in relative terms the improvement is moderate. It is most pronounced in the upper quartile of PI lengths: the Q3 and maximal PI lengths are reduced from (8.71%; 21.18%) to (6.48%; 15.07%), respectively. While it is advantageous to take into account the preparation kit and variant caller effects, the model remains fairly useful even if we leave them out.

### Fine-tuning the variance part of the model results in visible improvement

Our original goal is to see whether a useful PPV prediction is attainable based just on the inputs available in a typical VCF file. Therefore, we go back to the model (11, 1) and try to see whether it is possible to make it more useful by assuming that the variance parameter, σ, is dependent on the covariates (formula (4)). As a result, we arrive at model (11, 6) reported in the last line of Table 1, with the regression coefficients and p-values reported in Table 2.

**Table 2:** Regression coefficients for model (11, 6) from Table 1.

Mean part, μ	Estimate	Std error	t-value	p-value
Intercept	5.02E+000	2.64E-001	19.002	2.00E-016
WES_Indicator	-1.42E+000	2.93E-001	-4.847	1.68E-006
Ti/Tv	5.15E+000	1.76E-001	29.297	2.00E-016
MedDp	-1.96E-002	4.38E-003	-4.479	9.36E-006
DpLt5	-1.79E+000	8.78E-001	-2.042	0.0417
Ti/Tv * Ti/Tv	-1.75E-001	8.55E-002	-2.042	0.0417
MedDp * MedDp	-1.40E-004	1.32E-005	-10.58	2.00E-016
WES_Indicator * Ti/Tv	-4.47E+000	2.01E-001	-22.21	2.00E-016
WES_Indicator * MedDp	4.72E-002	5.90E-003	7.992	9.56E-015
WES_Indicator * DpLt5	1.76E+000	8.78E-001	2.009	0.0451
TiTv * MedDp	-1.91E-002	2.85E-003	-6.695	5.91E-011
**Variance part, σ**				
Intercept	-6.81047	0.3941	-17.28	2.00E-016
WES_Indicator	3.00998	0.38291	7.861	2.40E-014
Ti/Tv	-10.04787	1.33015	-7.554	2.05E-013
DpLt5	-0.33822	0.08178	-4.136	4.16E-005
Ti/Tv * Ti/Tv	-6.32795	0.76588	-8.262	1.31E-015
WES_Indicator * Ti/Tv	9.01236	1.95761	4.604	5.28E-006

According to formula (2), if σ includes only the intercept term, then the variance of PPV (and, consequently, the PI length) is dependent on the covariates only through the value of μ. If in reality σ is dependent on the covariates as well, then in model (11, 1) the prediction intervals will be too narrow (wide) for the points where σ is above (below) its average value. If we allow σ to depend on the covariates, we are able to obtain a better fit in that sense, which in many cases results in shorter prediction intervals. We see that happening in our last model (11, 6): compared to (11, 1) the five-number summary of PI lengths is improved except for the maximal PI length that increases from 21.18% to 44.45%.

## Discussion

As of today, the variant call set quality is routinely assessed by comparing the call set Ti/Tv ratio with a certain hard threshold. In this paper, we investigate whether it is possible to extend that simple rule and build a more advanced model that could provide a reasonably accurate quality estimate. We focus on estimating the proportion of true variants in a call set obtained from a human SNV study. Our main goal is to see whether a model that is based only on the common statistics found in a typical VCF file can be accurate enough.

We employ three gold standard datasets that span both WGS and WES studies. Since for those data sets the variants are known in advance and the counts of true and false SNV calls can be measured directly, we use that information to train the model. We measure the model performance in practical terms by looking at the prediction intervals for the estimated quantity, proportion of true variants.

Our first conclusion is that, if Ti/Tv and other commonly available predictors are already in the model then including Het/Hom ratio does not result in any notable improvement. That is partially due to a fairly strong negative association between Ti/Tv and Het/Hom that we can observe explicitly in Fig 4.

Next, we see that even though taking into account the preparation kit type and the type of variant caller is advantageous, it does not result in a dramatic performance improvement. Even if we do not take those factors into account, we are still able to have decent accuracy most of the time, especially if we fine-tune the variance part of the model. If we compare the performance of models (11, 6) and (19, 1) we see that the first four statistics for (11, 6) are on the par or even slightly better than those of model (19, 1).

That being said, it is fairly clear that building a variant-caller specific model can significantly improve the accuracy. The reason for that is twofold. First, our own results suggest that including variant caller type as a categorical factor is advantageous. Most importantly, each variant caller has its own set of quality statistics that can be used as predictors. It is hard to do so if the data are pooled across different variant callers because different callers report different quality statistics.

The variant quality score, QUAL, is the most obvious example. It is a good question how much accuracy we were to gain if we used QUAL as a covariate. Further, if we were to focus on Samtools, we could use such metrics as MQ, VDB, SGB, MQ0F, and the four p-values that reflect the strand, baseQ, mapQ, and tail distance biases. We could investigate which of those numerous quality statistics are the most useful. We could also see which metrics are useless or redundant and therefore do not have to be generated at all. Under the most optimistic scenario, it might turn out that using just QUAL results in a level of accuracy that is so high that none of other predictors are required and the same regression coefficients work well for both WES and WGS.

Researchers who develop variant calling applications could employ the methodology outlined in this paper for a similar purpose. That would also allow them to supply their software with an accurate PPV estimation tool that would be of great advantage to the end user.

## Supporting information

S1 Dataset Raw input data. Initial input data for Beta-binomial regression, including outliers.
(TXT)

S2 Dataset Processed input data. Input data for Beta-binomial regression where outlier(s) have been deleted, a few more observations have been added to cover the gaps in the covariate space, and centered and second order terms have been added.
(TXT)

S1 File Analysis code in R. R code to reproduce Figs 1–8 and Tables 1 and 2.
(R) Click here for additional data file.
